# Effects of classic psychedelic drugs on turbulent signatures in brain dynamics

**DOI:** 10.1162/netn_a_00250

**Published:** 2022-10-01

**Authors:** Josephine Cruzat, Yonatan Sanz Perl, Anira Escrichs, Jakub Vohryzek, Christopher Timmermann, Leor Roseman, Andrea I. Luppi, Agustin Ibañez, David Nutt, Robin Carhart-Harris, Enzo Tagliazucchi, Gustavo Deco, Morten L. Kringelbach

**Affiliations:** Latin American Brain Health Institute (BrainLat), Universidad Adolfo Ibañez, Santiago, Chile; Computational Neuroscience Group, Center for Brain and Cognition, Department of Information and Communication Technologies, Universitat Pompeu Fabra, Barcelona, Catalonia, Spain; Centre for Eudaimonia and Human Flourishing, Linacre College, University of Oxford, Oxford, United Kingdom; Centre for Psychedelic Research, Division of Psychiatry, Department of Brain Sciences, Imperial College London, London, United Kingdom; Division of Anaesthesia, School of Clinical Medicine, University of Cambridge, Cambridge, United Kingdom; Department of Clinical Neurosciences, University of Cambridge, Cambridge, United Kingdom; Leverhulme Centre for the Future of Intelligence, University of Cambridge, Cambridge, United Kingdom; The Alan Turing Institute, London, United Kingdom; Cognitive Neuroscience Center (CNC), Universidad de San Andrés, and CONICET, Buenos Aires, Argentina; Global Brain Health Institute (GBHI), University of California San Francisco (UCSF), San Francisco, CA, USA, and Trinity College Dublin (TCD), Dublin, Ireland; Psychedelics Division–Neuroscape, Department of Neurology, University of California San Francisco, San Francisco, CA, USA; Physics Department, University of Buenos Aires, and Buenos Aires Physics Institute, Buenos Aires, Argentina; Institució Catalana de la Recerca i Estudis Avancats (ICREA), Barcelona, Spain; Department of Neuropsychology, Max Planck Institute for Human Cognitive and Brain Sciences, Leipzig, Germany; School of Psychological Sciences, Turner Institute for Brain and Mental Health, Monash University, Melbourne, VIC, Australia; Department of Psychiatry, University of Oxford, Oxford, United Kingdom; Center for Music in the Brain, Department of Clinical Medicine, Aarhus University, Denmark

**Keywords:** Psychedelics, Psilocybin, LSD, Turbulence

## Abstract

Psychedelic drugs show promise as safe and effective treatments for neuropsychiatric disorders, yet their mechanisms of action are not fully understood. A fundamental hypothesis is that psychedelics work by dose-dependently changing the functional hierarchy of brain dynamics, but it is unclear whether different psychedelics act similarly. Here, we investigated the changes in the brain’s functional hierarchy associated with two different psychedelics (LSD and psilocybin). Using a novel turbulence framework, we were able to determine the vorticity, that is, the local level of synchronization, that allowed us to extend the standard global time-based measure of metastability to become a local-based measure of both space and time. This framework produced detailed signatures of turbulence-based hierarchical change for each psychedelic drug, revealing consistent and discriminate effects on a higher level network, that is, the default mode network. Overall, our findings directly support a prior hypothesis that psychedelics modulate (i.e., “compress”) the functional hierarchy and provide a quantification of these changes for two different psychedelics. Implications for therapeutic applications of psychedelics are discussed.

## Author Summary

Significant progress has been made in understanding the effects of psychedelics on brain function. One of the main hypotheses is that psychedelics work by changing the functional hierarchy of brain dynamics in a dose-dependent manner, modulating the encoding of the precision of priors, beliefs, or assumptions in the brain. We used a novel turbulence framework to investigate the changes in the brain’s functional hierarchy associated with two different psychedelics (LSD and psilocybin). This framework produced detailed signatures of turbulence-based hierarchical change for each psychedelic drug, revealing consistent and discriminate effects on a higher level network, that is, the default mode network.

## INTRODUCTION

In recent years, psychedelic (mind-manifesting) drug research has made a strong comeback (Nichols, 2016; Pollan, 2019), promising to deliver effective and safe treatments for neuropsychiatric disorders such as treatment-resistant depression (Carhart-Harris et al., 2021; Carhart-Harris, Bolstridge, et al., 2016) and addiction (Bogenschutz et al., 2015; Johnson, Garcia-Romeu, & Griffiths, 2017). Research so far indicates that the potential benefits far outweigh the risks (Johnson, Griffiths, Hendricks, & Henningfield, 2018; Johnson, Hendricks, Barrett, & Griffiths, 2019). Yet, a deeper knowledge of how psychedelics function in the human brain is needed to ensure best use of these compounds.

Here, we use a novel framework to directly characterize the effects of psychedelics on turbulent signatures in brain dynamics and gain insights into one of the leading hypotheses of how psychedelics function, namely that they work by relaxing hierarchical processing in the brain. This has been expressed in the REBUS (RElaxed Beliefs Under pSychedelics) and the anarchic brain hypothesis, which integrates Friston’s free-energy principle (Friston, 2010) with Carhart-Harris’s entropic brain hypothesis (Carhart-Harris, 2018). The authors hypothesize that psychedelics bring about a relaxation of the precision of high-level priors or “beliefs” (REBUS), allowing (anarchic) bottom-up information flow.

At the heart of this hypothesis is the idea that psychedelics modulate the encoding of the precision of priors, beliefs, or assumptions in the brain. This effect could manifest in various ways, including an alteration (i.e., a reduction) in the power of canonical brain rhythms, and the integrity of large-scale networks. There is compelling evidence that the action of classic psychedelics begins with agonism of 5-HT2A receptors (Nichols, 2016). A key functional effect of 5-HT2A receptor agonism via psychedelics is an increase in the sensitivity of excitatory neurons expressing the receptor (e.g., deep-layer pyramidal neurons) resulting in a spike-wave-decoupling and dysregulation of spontaneous population-level activity. Mapping these effects to subjective phenomena is a work in progress; however, it is tempting to speculate that subjective phenomena like ego dissolution (Milliere, 2017; Nour, Evans, Nutt, & Carhart-Harris, 2016), unitive experiences (Griffiths et al., 2016; Roseman, Nutt, & Carhart-Harris, 2018), and a sense of the ineffable (Pollan, 2019) relate to the relaxation (or “breakdown”) of particularly core or high-level priors or beliefs, encoded via the functioning of high-level systems in the brain.

The present paper uses a novel turbulence framework to determine the functional hierarchy of any brain state, by calculating measures of information processing in the human brain inspired by turbulence theory (Kolmogorov, 1941b; Kuramoto, 1984) but applied to neuroscience (Sheremet, Qin, Kennedy, Zhou, & Maurer, 2019). Long-standing research in fluid dynamics has shown that turbulence facilitates the rapid transfer of energy through fluids (Frisch, 1995; Kolmogorov, 1941b). In terms of brain dynamics, the turbulent core is determined by the local synchronization between brain areas that, in turn, are linked to the rotational vortices found in fluid dynamics. As such, turbulence has been demonstrated in large-scale neuroimaging data from healthy participants scanned with functional magnetic resonance imaging (fMRI) (Deco & Kringelbach, 2020) and ensures efficient information transfer (Deco, Kemp, & Kringelbach, 2021; Deco & Kringelbach, 2020). The size of the turbulent vortices is what defines the different information-processing scales, and it has also been shown how rare long-range connections enhance information processing throughout the brain (Deco, Perl, et al., 2021; Escrichs et al., 2021).

In order to determine the psychedelic-specific changes to the functional hierarchy, our framework employed two complementary model-free and model-based approaches inspired by the turbulence theory of physics (Frisch, 1995; Kolmogorov, 1941b; Kuramoto, 1984). Using the model-free approach, we measured information transmission across space and timescales using four novel and distinct measures recently introduced by Deco and Kringelbach (2020): turbulence, information transfer, information cascade flow, and information cascade. The model-based approach used a whole-brain model to mechanistically explain the statistical dependencies of the neuronal dynamics observed in the empirical neuroimaging data (Deco, Cabral, et al., 2017; Deco & Kringelbach, 2020; Deco, Kringelbach, Jirsa, & Ritter, 2017). The model allowed us to study simulated perturbation-elicited changes in global and local brain activity to assess the system’s susceptibility and information-encoding capability, offering new insights into the brain’s reactivity to external perturbations, such as perturbations induced pharmacologically or by electrical stimulations.

We were able to demonstrate that this framework differentiates between the different psychedelics in terms of providing a specific signature of the way they perturb the hierarchy of brain dynamics. Overall, this provides novel information on how different psychedelics work.

## RESULTS

**Figure 1.**
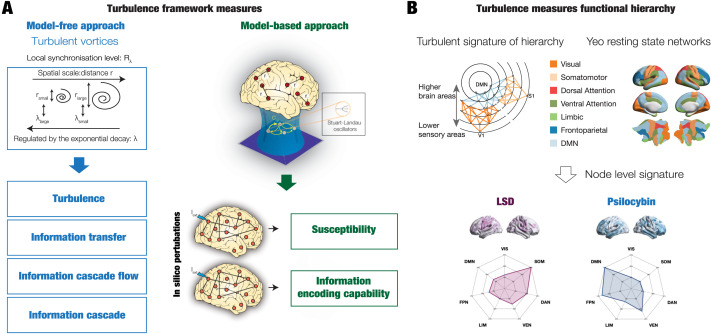
Determining the functional hierarchy in brain dynamics using a turbulence framework. (A) The turbulence framework uses model-free and model-based measures. The left panels show a schematic of how measures of turbulence, information transfer, information cascade flow, and information cascade are obtained from estimating the local level of synchronization characterized by the Kuramoto local order parameter (*R*; see the Methods section) at different scales. The right panel shows a Hopf whole-brain model that describes the dynamics of each brain area through a Stuart Landau nonlinear oscillator. The system of local oscillators is connected through the anatomical connectivity to simulate the global dynamics induced by each psychedelic. Whole-brain modeling allows us to obtain measures that arise from the perturbative approach. We simulated external perturbations and evaluated the model’s response for each condition’s brain state by quantifying the susceptibility and information capacity measures. (B) The brain is hierarchically organized such that information flows from lower sensory regions to higher association regions (Smallwood et al., 2021). The top left panel sketches how information comes into primary visual (V1) and somatosensory (S1) regions and flows through the distinct visual (orange) and somatomotor (yellow) networks before reaching the default mode network (DMN, light blue). The top right panel renders the seven Yeo resting-state networks on the human brain, which forms the basis for the hierarchical measure obtained by using the turbulence framework on the two different psychedelics (LSD and psilocybin). The brain plots show the resulting node-level turbulence for each psychedelic drug. The spider plots at the bottom show how this gives rise to different signatures of the degree of participation of the Yeo resting-state networks for each psychedelic.

We used a novel turbulence framework (Deco & Kringelbach, 2020) to investigate changes in brain hierarchy induced by different psychedelic drugs measured with fMRI in healthy volunteers from two different datasets. Figure 1 provides a schematized version of the general framework used to determine the functional hierarchy in brain dynamics.

Participants received intravenous injections of lysergic acid diethylamide (LSD) (*N* = 15) or psilocybin (*N* = 9), and placebo in two separate studies (Carhart-Harris, Muthukumaraswamy, et al., 2016; Carhart-Harris et al., 2012). Experimental designs differed but all were placebo controlled (saline injection), with participants blinded to condition. Here we focused on the effects of psychedelics on the brain’s turbulent dynamics based on the recent demonstration that the brain exhibits a turbulent dynamic intrinsic backbone that facilitates large-scale network communication (Deco & Kringelbach, 2020).

Within the model-free framework, we studied changes in information transmission flow across spatial and temporal scales using four distinct measures: turbulence, information transfer, information cascade flow, and information cascade. These analyses were performed based on the Kuramoto order parameter describing the local degree of synchronization of a brain region, *n*, as a function of space, $\bar{x}$, and time *t*, at a given scale *λ*. The scale of the local synchronization is defined by the parameter *λ*, which determines the spatial distance where the synchronization is assessed. We explored scales ranging from 0.01 (∼100 mm) to 0.21 (∼5 mm), in steps of 0.03, where high values of *λ* denote short distances in the brain and vice versa.

**Figure 2.**
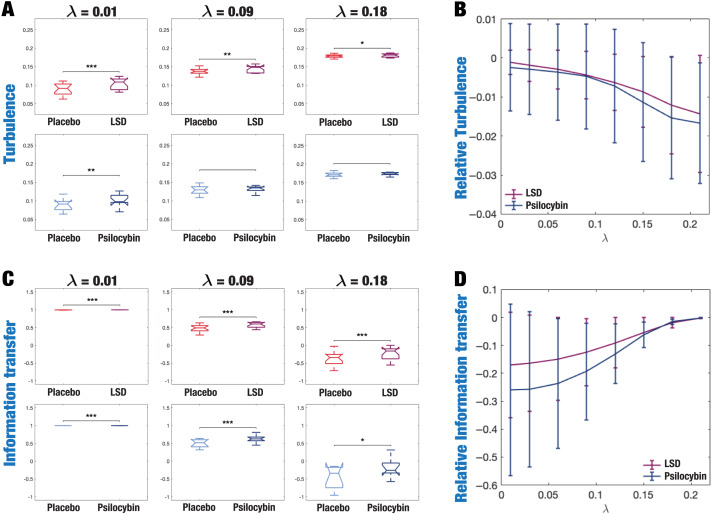
Turbulence and information transfer changes across scales under the psychedelic state. Within the model-free framework, we investigated the degree to which psychedelics modulate information processing at the whole-brain level, characterized by turbulence and information transfer measurements. (A) Turbulence estimates the level of local synchronization at different spatial scales by measuring the spatiotemporal variability of the local Kuramoto order parameter. We calculated the turbulence for spatial scales (*λ*) ranging from 0.01 to 0.21 in steps of 0.03. Both psychedelics, LSD (above) and psilocybin (below), increase the level of turbulence compared with placebo, particularly at larger spatial scales (smaller *λ*). The effect size between statistically significant results was assessed by Cohen’s *d*. *D* values for LSD, respectively: *d* = 0.9441 (*λ* = 0.01), *d* = 0.7013 (*λ* = 0.09), *d* = 0.4237 (*λ* = 0.18). *D* values for psilocybin, respectively: *d* = 0.8034 (*λ* = 0.01), *d* = 0.5230 (*λ* = 0.09), *d* = 0.4451 (*λ* = 0.18). (B) Complementarily, we computed a linear fitting of the mean level of turbulence at each spatial scale. The panel shows the slope obtained as a function of *λ*, and the vertical lines represent the standard deviation across subjects. The two slopes show a similar pattern, although they slightly differ by showing at higher spatial scales (lower *λ*). (C) The spatial information transfer measurement informs how the information travels across space at a specific scale (*λ*). This measure is defined as the slope of a linear fitting in log-log scale of the time correlation between the Kuramoto local order parameter of two brain areas at the same scale as a function of its Euclidean distance (*r*) within the inertial subrange. Psychedelics significantly increase information transfer at all spatial scales. The effect size between statistically significant results is clear. *D* values for LSD, respectively: *d* = 1.0332 (*λ* = 0.01), *d* = 1.0281 (*λ* = 0.09), *d* = 0.8809 (*λ* = 0.18). *D* values for psilocybin, respectively: *d* = 0.7920 (*λ* = 0.01), *d* = 1.2115 (*λ* = 0.09), *d* = 0.9206 (*λ* = 0.18). (D) Following the same procedure as in panel B, we computed the linear fit of the information transfer measure. As can be seen, psilocybin differs from the LSD slope patterns, showing a much steeper slope-scale, denoting higher sensitivity to differences in the degree of transmission of information across scales. The vertical lines represent the standard deviation across subjects. * Indicates *p* < 0.05, ** indicates *p* < 0.01, and *** indicates *p* < 0.001.

Compared with placebo, psychedelics induce significant increases in turbulence (Figure 2A). In the LSD condition, these increases were found across all spatial scales but were more pronounced at the longer ones. In contrast, the effects in psilocybin were observed only at longer distances in the brain (*λ* < 0.06). Complementarily, we calculated a linear fit to the mean turbulence at each *λ* and captured the slopes of each condition representing its level of turbulence at each spatial scale. Figure 2B resumes the measures of information transmission through time and space calculated by changes in turbulence on each different scale. Both psychedelics monotonically decrease the slope at longer spatial scales.

We computed the information transfer measure, which denotes how information travels across space at a given spatial scale. We found that psychedelics significantly increase information transfer across all spatial scales in the brain, favoring information transmission. Figure 2C shows boxplots of the statistically significant differences between conditions that passed the permutation-based paired *t* test with *p* < 0.05. We performed the exact computations presented in Figure 2B, but now for the information cascade measurement. Figure 2D summarizes the behavior of this measure at different spatial scales. LSD and psilocybin present a similar slope-scale relationship at shorter spatial scales (larger lambdas), while their differ at the shorter ones suggesting that psilocybin is more sensitive to differences in the degree of information transmission across scales.

**Figure 3.**
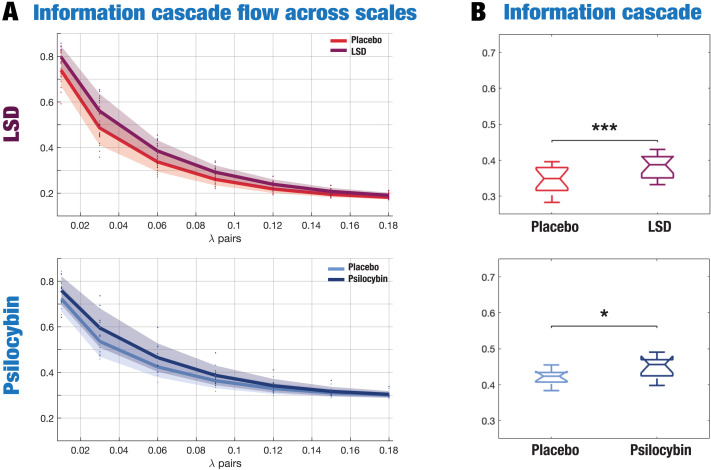
Information flow across scales and information cascade changes under the psychedelic state. (A) The information flow across scales outlines the degree of predictability of the synchronization level at a certain scale (*λ*) given by the precedent scale (*λ* − Δ*λ*), where Δ*λ* is the discretization of the scale. LSD significantly increases information flow at all spatial scales, while psilocybin does so only at larger spatial scales (*λ* < 0.09). (B) Similarly, the information cascade flow, defined as the average information flow across scales, significantly increases under the psychedelic state, for both LSD and psilocybin, compared with placebo. * Indicates *p* < 0.05, ** indicates *p* < 0.01, and *** indicates *p* < 0.001. *D* values for LSD: *d* = 1.0437; and psilocybin: *d* = 0.6566.

We then characterized the transmission of information across spatial and temporal scales using the information cascade flow measurement described as the correlation of the signal at a given scale *λ*, with the signal at a lower scale *λ* − Δ*λ*, in consecutive time steps, *t* + Δ*t*. As shown in Figure 3A, we found that LSD significantly increased information cascade flow at all *λ* scales compared with placebo, while psilocybin also did so at higher spatial scales, that is, lower *λ* denoting long distances in the brain. Finally, we found evidence of significantly increased information cascade under the psychedelic state compared with placebo (Figure 3B). This measure characterizes the global degree of information transmission across scales and is obtained by averaging the information cascade flow across all *λ* scales.

The increases in turbulence, information transfer, and information cascade observed under LSD did not correlate with the subjective experience reports. For psilocybin, we observed that only the increases in information cascade correlated with ratings on ego-dissolution (see the Supporting Information, Figures S1–S3).

**Figure 4.**
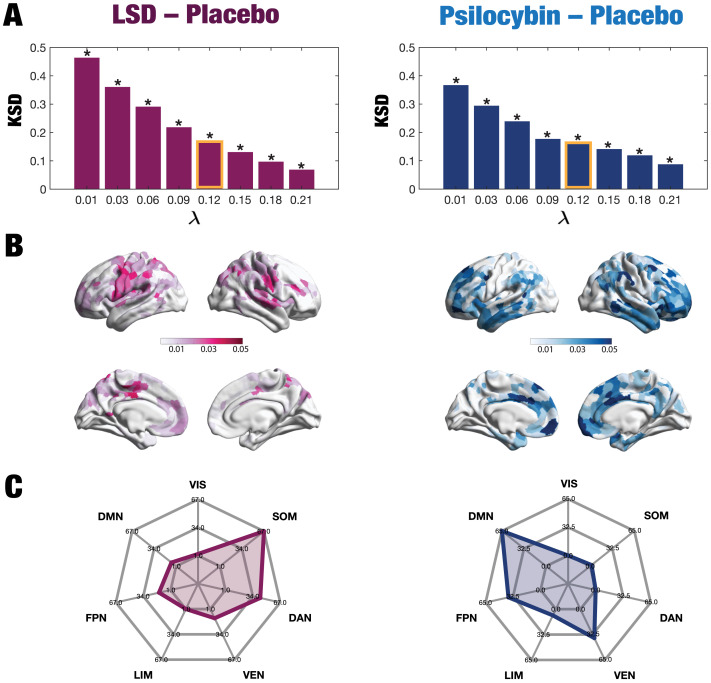
Node-level turbulence. We computed the node variability of the local synchronization defined as the standard deviation across time of the local Kuramoto order parameter. (A) The panels plot the Kolmogorov–Smirnov distance (KSD) quantifying the difference between the distributions of the node-level turbulence for each psychedelic and placebo, at each spatial scale. The smaller the KSD values, the more similar the distributions; therefore, the greatest differences were found at larger spatial scales (lower *λ* values). (B) The brain schemes show the absolute difference of the node-level turbulence for *λ* = 0.12 as an example (shown in yellow in panel A). (C) We then picked the upper 15% quantile of the absolute difference of the node-level turbulence between conditions, indexed the resting-state network to which they belong, according to Yeo’s resting-state network, and estimated the number of nodes per network. The spiderweb charts outline the number of nodes in the higher 15% quantile of the absolute difference for each comparison and network (VIS: visual; SOM: somatomotor; DAN: dorsal attention network; VEN: ventral network; LIM: limbic; FPN: frontoparietal network; DMN: default mode network). The networks that showed the most significant differences between LSD and placebo are the SOM and DAN; and between psilocybin and placebo, the FPN, DMN, and VEN. The results generalize to the other spatial scales.

Furthermore, we calculated the node variability of the local synchronization defined as the standard deviation across time of the local Kuramoto order parameter for each condition. We calculated the similarity of the node-level turbulence between each psychedelic compound and the counterpart placebo using the Kolmogorov–Smirnov distance (KSD) between them at each spatial scale. Figure 4A shows that the difference between conditions is more prominent at lower *λ* scales for both psychedelic drugs. For each condition, we then computed the absolute difference of the node-level turbulence between the psychedelic state and the placebo at *λ* = 0.12. Figure 4B renders this absolute difference onto the brain’s cortex. Subsequently, we picked the nodes for each comparison of the upper 15% quantile, identified the resting-state network to which they belong, and counted the number of nodes per network. This strategy allowed us to describe the precise signature of turbulence-based hierarchical change for each psychedelic drug, revealing how each one impacts the dynamics of the networks. As can be seen in Figure 4C, differences between LSD and placebo were mainly found in the somatomotor (SOM) and dorsal attention networks (DAN), and between psilocybin and placebo in the default mode network (DMN), frontoparietal (FPN), and ventral network (VEN).

**Figure 5.**
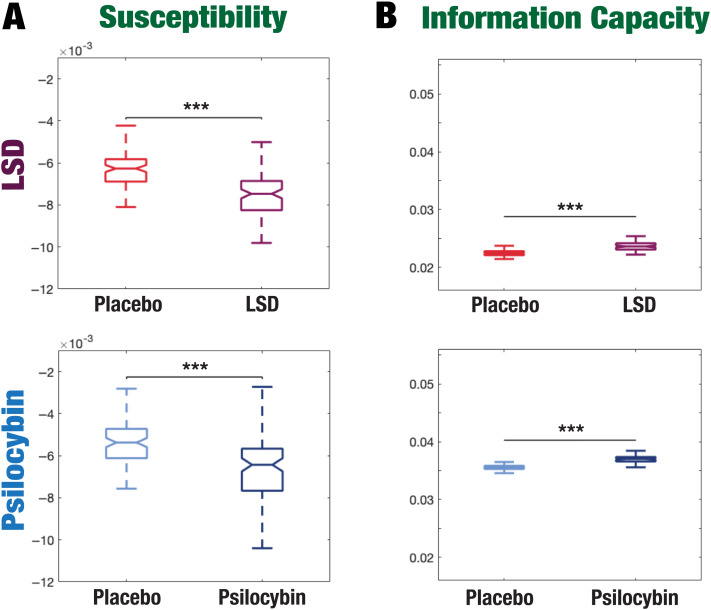
Model-based susceptibility and information capacity changes under the psychedelic state. We used a model-based approach to assess the effects of external perturbations on brain dynamics. (A) We quantified the susceptibility measure, which estimates the responsiveness of the in silico whole-brain dynamics to external perturbations. LSD and psilocybin significantly decrease the susceptibility level compared with placebo. *D* values for LSD and psilocybin, respectively: *d* = 1.2522, *d* = 0.8336. (B) The information capacity measurement denotes the encoding capability of the whole-brain model. In other words, this appraisal captures how the simulated external perturbations are encoded in brain dynamics. Both psychedelic drugs significantly increase the information-encoding capacity. *D* values for LSD and psilocybin, respectively: *d* = 1.400, *d* = 2.5615. *P* values were assessed using the Wilcoxon rank sum test and corrected for multiple comparisons. * Indicates *p* < 0.05, ** indicates *p* < 0.01, and *** indicates *p* < 0.001.

We applied a whole-brain computational modeling approach based on the sensitivity of these models to react to external in silico perturbations and their capability to offer insights into the mechanisms underlying the global complexity and dynamical stability of brain activity (Deco et al., 2018; Jobst et al., 2021) (see the Methods section). For each of the four brain states (LSD vs. placebo, and psilocybin vs. placebo), we constructed a whole-brain dynamical model based on the normal form of a supercritical Hopf bifurcation (Deco, Kringelbach, et al., 2017). The model was coupled to the structural connectivity obtained through diffusion tensor imaging and used the exponential distance rule of anatomical connections as a cost-of-wiring principle (Ercsey-Ravasz et al., 2013; Markov et al., 2013; Markov et al., 2014). Each model was fitted to optimally reproduce the empirical spatiotemporal dependencies of each brain state characterized using the functional connectivity (FC) measure as a function of the global coupling parameter *G*. For each *G* and condition, we performed 100 simulations and calculated the fitting performance as the Euclidean distance between the empirical and the simulated FC. The minimum distance between the empirical and the simulated FC defines the optimal operating point of the model. Then, to evaluate how each model-based brain state reacts to external stimuli, we systematically applied in silico perturbations and quantified the functional consequences of these perturbations using the susceptibility and information capability measures. Perturbations were implemented by randomly changing the bifurcation parameter of each brain area *a*_*n*_ in the model within the range [−0.02:0]. The susceptibility measure reflects the sensitivity of the whole-brain model to react to external perturbations. It is defined by the difference between the perturbed and unperturbed mean of the modulus of the local order parameter across time ($\tilde{R}$_*λ*_*s*__($\bar{x}$, *t*) and *R*_*λ*_*s*__($\bar{x}$, *t*), respectively), averaged over all brain nodes. Complementarily, the information capability denotes the capacity of the brain to encode external perturbations in brain dynamics and is defined as the standard deviation across trials of the susceptibility measure. Figure 5A shows that LSD and psilocybin significantly decrease susceptibility. Complementarily, the information-encoding capacity significantly increases following the psychedelic drug administration, compared with placebo (*p* < 0.001, two-sided Wilcoxon rank sum test). The results for both measures were estimated at *λ* = 0.18.

## DISCUSSION

Here we used two independent fMRI datasets of healthy participants receiving moderate to high doses of LSD or psilocybin in placebo-controlled designs to determine the psychedelic-specific modulation of the brain’s dynamic functional hierarchy and assess its impact on information processing. Our framework comprised model-free and model-based approaches inspired by signatures of turbulence in complex systems. Using this approach, we aimed to test a leading hypothesis of the brain action of psychedelics, that is, that they relax the properties of canonical systems in the brain—encoding internal models or assumptions. Results revealed generally consistent increases in turbulent signatures across the psychedelics.

Serotonin 2A receptor agonism is a defining pharmacological property of the “classic” psychedelics—and LSD and psilocybin are perhaps the most familiar examples. At the population and systems level, psychedelics have been shown to increase the entropy of spontaneous brain activity (Carhart-Harris et al., 2014; Herzog et al., 2020), broaden the repertoire of connectivity states (Atasoy, Vohryzek, Deco, Carhart-Harris, & Kringelbach, 2018; Tagliazucchi, Carhart-Harris, Leech, Nutt, & Chialvo, 2014; Varley, Carhart-Harris, Roseman, Menon, & Stamatakis, 2020), and enhance global connectivity between high-level networks and the rest of the brain (Carhart-Harris, Muthukumaraswamy, et al., 2016; Tagliazucchi et al., 2016). The merit of turbulence-related metrics, however, is the information they confer about information transfer in the brain and their relevance to complexity science—which offers a rich and developing language for understanding the properties of complex systems.

Using a model-free approach, we first explored the global information transmission characteristics. This approach is grounded in the innovative and sophisticated framework for analyzing whole-brain dynamics, recently developed by Deco and Kringelbach (2020), through which they revealed that the healthy human brain shows turbulent dynamics (Frisch, 1995; Kolmogorov, 1941b; Kuramoto, 1984). Summarizing the results, compared with placebo, both psychedelics promoted greater information transmission in the brain, in both the spatial and the temporal domains. Specifically, significant increases were observed in turbulence, information transfer across scales, information cascade flow, and information cascade. The present framework could equally well be described in terms of the local Kuramoto order parameter and its use for characterizing information transmission, that is, as a generalization of the concept of metastability in neuroscience, pioneered by Shanahan and Kuramoto (Kuramoto, 1984; Wildie & Shanahan, 2012). Nevertheless, such a description does not capture the generality of the principles governing brain dynamics and most, if not all, physical systems. The turbulence framework offers a characterization of the dynamics underlying different brain states and provides a principled, mechanistic way to describe information transmission across spacetime.

Thus, irrespective of the type of psychedelic, the psychedelic state appears to show a clear trend towards a greater transmission of information—characterized by increases in turbulence and information transfer values—through long-range spatial scales, that is, smaller lambdas. Long-range cortical connections have long been thought to be crucial for the emergence of consciousness. In fact, it has been shown that long-range connections are associated with enhanced cognitive processing in healthy participants (Deco, Perl, et al., 2021), whereas a decrease in global information processing and large-scale functional connectivity appears to be a robust signature of reduced conscious levels (Bourdillon et al., 2020; King et al., 2013; Vatansever et al., 2020). These findings suggest that the recruitment of long-distance connections plays a fundamental role in the flow of information through the cortex, allowing communication between different brain areas and ultimately supporting the emergence of conscious awareness. It is tempting to speculate that increased long-range information transfer under psychedelics relates to their purported ability to facilitate psychological insight (Yaden & Griffiths, 2020)—consistent with the etymology of the term “psychedelic”—that is, “mind-revealing” (Hartogsohn, 2018; Nichols, 2016; Osmond, 1957).

We explored node-level changes in turbulence to identify the primary brain areas driving the turbulent dynamic core and thus involved in changing the whole-brain dynamics underlying each psychedelic state. Noteworthily, we found that brain regions belonging to the somatomotor and dorsal attention network showed the most critical differences in the LSD-induced brain state; and for psilocybin the default mode, frontoparietal and ventral networks showed the greatest differences. These results highlight the specific functional changes to the functional hierarchy, revealing that psilocybin directly impacts high-level networks, whereas LSD has stronger effects on primary visual-sensory areas. Given that the level of turbulence is increased for both LSD and psilocybin at the different spatial scales, we speculate that changes in the setting such as music may increase turbulence and therefore increase the observed effect. However, this is out of the scope of the present paper and deserves thorough investigation.

Furthermore, we used a model-based approach to investigate how whole-brain dynamics underlying each brain state impact the brain’s capacity to encode external perturbations. To do so, we built a whole-brain model based on the normal form of a supercritical Hopf bifurcation (Deco, Kringelbach, et al., 2017), simulating the empirical fMRI statistical dependencies. We applied in silico external perturbations to evaluate how each model-based brain state reacts to (simulated) external perturbations. We found that the brain is less sensitive to external perturbations under psychedelics as shown by the susceptibility measure. Conversely, consistent increases in the brain’s information-encoding capacity were observed across all psychedelics, suggesting that the brain becomes more selective and enhances specificity when dealing with information processing.

It is important to note that the susceptibility measure is not a measure of complexity but rather a measure of the ability of a system to be perturbed, and it is also not a measure of the ensuing complexity of the response (as in TMS-PCI studies). In contrast, the information capacity measures the ability of the system to encode external inputs, and as such is more closely related to complexity measures such as Lempel-Ziv-Welch (LZW), automatic complexity evaluator (ACE), and synchrony coalition entropy (SCE) (used and defined by Schartner et al., 2015).

First proposed by Massimini et al. (2005), the perturbative approach has been increasingly applied to investigate brain function because of its potential to relate local neural activity changes to global brain dynamics and reveal the underlying detailed causal mechanisms. This approach is a suitable complement to observational approaches that are typically descriptive and correlative and that thus do not offer insights into the underlying mechanisms. Data-constrained whole-brain models are key to studying perturbation-induced changes in neural activity, as they allow parameter optimization and targeted perturbation to be systematically explored in different brain regions. Perturbations in dynamical models of whole-brain activity have been shown to dissociate different brain states (Deco et al., 2018; Jobst et al., 2021; Sanz Perl et al., 2021), providing a robust metric and computational tool capable of characterizing and unraveling brain states. While a previous study compared perturbation-induced changes in brain dynamics in the LSD state versus placebo using a similar model-based approach (Jobst et al., 2021), it did not focus on the information-processing characteristics of brain states. Still, as in the present study, this previous one did show greater variability in the perturbational responses in the LSD condition compared with placebo (Jobst et al., 2021). This variability refers to what we call here information-encoding capability, a measure that captures how the external perturbations are encoded in whole-brain dynamics. The previous study also reported that brain dynamics under LSD took longer to recover to baseline activity after perturbation.

Psychedelic drugs appear to broaden the brain’s dynamical repertoire (Atasoy et al., 2017; Lord et al., 2019; Tagliazucchi et al., 2014) and enhance global functional connectivity by shifting the brain’s global working point towards a more globally connected profile (Preller et al., 2018; Tagliazucchi et al., 2016). The present study’s findings are consistent with this characterization and advance on it by suggesting that psychedelics also promote a greater spread of neural activity and an associated increase in information transfer and “mixing” across domains and scales throughout the brain.

Taken together, our findings show that psychedelics exhibit a particular pattern of (generally increased) turbulent dynamics that may relate to their characteristic effects on conscious experience. While helping to enrich our understanding of the brain basis of the psychedelic state, these findings also deepen our understanding of the functional relevance of turbulence-related metrics in relation to brain function more broadly.

## METHODS

To quantify the functional hierarchy under the acute effects of different psychedelics (LSD and psilocybin), we applied a novel turbulence framework to two psychedelic datasets in human participants. We used the power of whole-brain model-free and model-based approaches to characterize the hierarchy for the different compounds. For the model-free approach, we estimated the levels of turbulence, information transfer, information cascade flow, and information cascade. For the model-based approach, we estimated the susceptibility and information-encoding capability measures. Below we briefly summarize the participants, study settings, data acquisition, and preprocessing protocols.

### Ethics Statement

The National Research Ethics Service Committee (NRES) London–West London approved both studies, which were conducted following the revised Declaration of Helsinki (2000), the International Committee on Harmonization Good Clinical Practice guidelines, and the National Health Service Research Governance Framework. Imperial College London sponsored the research carried under a Home Office license for research with schedule 1 drugs. All participants provided written informed consent prior to participation.

### Participants

#### LSD.

A complete description of the LSD study protocol can be found in the original paper (Carhart-Harris, Muthukumaraswamy, et al., 2016). Twenty healthy subjects (four females, mean age = 30.9 ± 7.8 years) recruited via word of mouth participated after study guidance and physical and mental health screening. The screening for physical health included an electrocardiogram, routine blood tests, and urine tests for recent drug use and pregnancy. All participants provided full disclosure of their drug use history in a psychiatric interview. Critical exclusion criteria included the following: <21 years of age, pregnancy, personal history of diagnosed psychiatric illness, immediate family history of a psychotic disorder, an absence of previous experience with a classic psychedelic drug (e.g., LSD, mescaline, psilocybin/magic mushrooms, or DMT/ayahuasca), any psychedelic drug use within six weeks of the first scanning day, pregnancy, problematic alcohol use (i.e., >40 units consumed per week), or a medically significant condition rendering the volunteer inadequate for the study. Participants attended two scanning sessions on different days at 8:00 (LSD and placebo) at least two weeks apart in balanced order, within-participants design. LSD (75 μg in 10 ml saline) or placebo (10 ml saline) was delivered as bolus injections over 2 min while participants were instructed to close their eyes and relax. Following an acclimatization period of 60 min inside a mock MRI scanner, three fMRI scans were conducted in the following order: eyes-closed resting state, rest while listening to music, and another eyes-closed resting-state session. Participants rated the Visual Analog Scale (VAS) via button press and a digital display screen presented after each scan. We report results for the pre- and post-music resting-state scans concatenated in time.

#### Psilocybin.

A complete description of the psilocybin study protocol can be found in the original paper (Carhart-Harris et al., 2012). In brief, 15 participants were included in the study following rigorous exclusion criteria: no younger than 21 years of age, pregnancy, history of psychiatric disorders, cardiovascular disease, substance dependence, claustrophobia, blood or needle phobia, or adverse response to hallucinogens. Furthermore, participants were excluded if their mean framewise displacement exceeded 0.4 mm. All participants underwent two 12-min eyes-closed resting-state fMRI scans over separate sessions, at least seven days apart. In each session, subjects were injected intravenously with either psilocybin (2 mg dissolved in 10 mL of saline, 60-s injection) or a placebo (10 mL of saline, 60-s injection) in a counterbalanced design. The injections were given manually by a medical doctor within the scanning suite. The infusions began precisely 6 min after the start of the 12-min scans and lasted 60 s. The subjective effects of psilocybin were felt almost immediately after injection and sustained for the remainder of the scanning session and were rated via button press using the VAS scale. All participants had previous experience with a hallucinogenic drug but not within six weeks of the study.

### MRI Data Acquisition

#### LSD.

Imaging was performed on a 3T GE HDx system. High-resolution anatomical images were acquired with 3D fast spoiled gradient echo scans in an axial orientation, with a field of view = 256 × 256 × 192 and matrix = 256 × 256 × 129 to yield 1-mm isotropic voxel resolution. TR/TE = 7.9/3.0 ms; inversion time = 450 ms; flip angle = 20. BOLD-weighted fMRI data were acquired using a gradient-echo planar imaging sequence, TR/TE = 2,000/35 ms, field of view = 220 mm, 64 × 64 acquisition matrix, parallel acceleration factor = 2, 90 flip angles. Thirty-five oblique axial slices were acquired interleaved, each 3.4 mm thick with zero slice gap (3.4-mm isotropic voxels). The precise length of each of the BOLD scans was 7 min 20 s. One subject aborted the experiment because of anxiety, and four others we excluded for excessive head motion in the scanner (defined as >15% of volumes with mean framewise displacement > 0.5 (Carhart-Harris, Muthukumaraswamy, et al., 2016), leaving 15 subjects with 434 TRs each for analysis.

#### Psilocybin.

Imaging acquisitions were identical to the LSD experiment with the following exceptions: BOLD-weighted fMRI data were acquired at TR/TE = 3,000/35 ms, field of view = 192 mm. Thirty-three oblique axial slices were acquired interleaved, each 3 mm thick with zero slice gap (3 × 3 × 3 mm voxels). Six participants were excluded for excessive motion, leaving nine participants with 97 TRs for analysis.

### Resting-State Preprocessing

We used the CONN toolbox, version 17f (CONN; https://www.nitrc.org/projects/conn; Whitfield-Gabrieli & Nieto-Castanon, 2012) based on Statistical Parametric Mapping 12 (https://www.fil.ion.ucl.ac.uk/spm), implemented in MATLAB to preprocess and denoise the LSD and psilocybin fMRI data. For each condition (placebo, LSD; placebo, psilocybin), we applied a standard pipeline including the following steps: removal of the first three volumes to eliminate saturation effects and achieve steady-state magnetization; functional realignment to correct for movement; slice-timing correction to account for variations in time of acquisition among slices; identification of outlier scans for subsequent scrubbing through the quality assurance/artifact rejection software art (https://www.nitrc.org/projects/artifact_detect), using the default CONN settings of five global signal Z-values and 0.9 mm for the identification of outlier volumes; normalization to Montreal Neurological Institute (MNI152) standard space with 2-mm isotropic resampling resolution, using the segmented gray matter image from each volunteer’s high-resolution T1-weighted image, together with an a priori gray matter template; spatial smoothing with a Gaussian kernel of 6 mm full width at half-maximum. For denoising details, see Luppi et al. (2021), Luppi et al. (2019), and Luppi et al. (2020).

### Tractography Analysis

Following Deco and Kringelbach (2020), we used the freely available Human Connectome Project (HCP) database comprising diffusion spectrum and T2-weighted neuroimaging data from 32 healthy subjects. A comprehensive account of the acquisition parameters can be found on the HCP website (Schaefer et al., 2018). The free Lead-DBS software package (https://www.lead-dbs.org/) provides the preprocessing described in detail in Horn, Neumann, Degen, Schneider, and Kühn (2017). Concisely, the data were processed using a q-sampling imaging algorithm implemented in DSI Studio (https://dsi-studio.labsolver.org). Segmentation of the T2-weighted anatomical images produced a white matter mask and coregistering the images to the b0 image of the diffusion data using SPM12. For each HCP participant, 200,000 fibers were sampled within the white matter mask. Fibers were transformed into MNI space using Lead-DBS (Horn & Blankenburg, 2016). Lastly, we adopted the standardized methods in Lead-DBS to obtain the structural connectomes from the Schaefer 1000 parcellation (Schaefer et al., 2018).

### Parcellation Scheme

We used the Schaefer atlas with 1,000 parcels (Schaefer et al., 2018) to set brain nodes and derive the corresponding time series. Also, we computed the Euclidean distances between the centers of gravity of the parcels in Schaefer 1000 parcellation in MNI space.

### Model-Free Framework

#### Turbulence.

The amplitude turbulence, *R*_*λ*_($\bar{x}$, *t*), is defined as the modulus of the Kuramoto local order parameter for a given brain node as a function of time:

$$R_{\lambda }\left(\bar{x},t\right)=\left\|k\int_{-\infty }^{\infty }d{\bar{x}}^{\prime G_{\lambda }}\left(\bar{x}-{\bar{x}}^{\prime }\right)e^{i\phi \left({\bar{x}}^{\prime },t\right)}\right\|,$$ (1)

where ‖
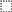
‖ is the modulus of the complex number, *G*_*λ*_ is the local weighting kernel *G*_*λ*_($\bar{x}$) = *e*^−*λ*∣$\bar{x}$∣^, *ϕ*($\bar{x}$, *t*) are the phases of the spatiotemporal data, k is the normalization factor ${\left[\int_{-\infty }^{\infty }d{\bar{x}}^{\prime }G_{\lambda }\left(\bar{x}-{\bar{x}}^{\prime }\right)\right]}^{-1}$, and *λ* defines the spatial scaling. Hence, *R*_*λ*_ represents the local levels of synchronization at a given scale, *λ*, as function of space, $\bar{x}$, and time, *t*. Inspired by the rotational vortices found in fluid dynamics, the turbulence measure characterizes the *brain vortex space*, *R*_*λ*_, over time. Note that *t* can take values from 1 to 434 for the LSD condition, and from 1 to 97 for the psilocybin condition; and $\bar{x}$ can take values from 0.2165 to 172.9464.

The level of amplitude turbulence, *D*_*λ*_, is defined as the standard deviation across time and space of the modulus of local Kuramoto order parameter (*R*_*λ*_).

$$D_{\lambda }={\langle {R_{\lambda }}^{2}\rangle }_{\bar{x},t}-{\langle R_{\lambda }\rangle }_{\bar{x},t}^{2},$$ (2)

where the brackets 〈
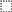
〉_$\bar{x}$,*t*_ denote averages across time and space.

#### Information transfer.

The spatial information transfer shows how the information travels across space at a specific scale, *λ*. This measure is defined as the slope of a linear fitting in log-log scale of the time correlation between the Kuramoto local order parameter of two brain areas, at the same scale as a function of its Euclidean distance (r) within the inertial subrange (the limited range where turbulence energy is transferred from larger to smaller scales without loss). We used the linear fit only to quantify the level of decay of the correlation of the local level of synchronization with distance. Please note that we compute the linear fit in a limited log-log space and, as such, we do not have a distribution, and thus are not trying to fit a power law.

$$\log\left(corr_{t}\left(R_{\lambda }\left(\bar{x}\right),R_{\lambda }\left(\bar{x}+r\right)\right)\right)=A_{\lambda }\times \log\left(r\right)+B_{\lambda },$$ (3)

where *corr*_*t*_ is the pairwise correlation across time, *A*_*λ*_ and *B*_*λ*_ are the fitting parameters for each scale (*λ*), where *r* is the spatial distance in the brain. The negative slope (*A*_*λ*_) stands for the transfer in the spatial direction *r* of the information in terms of time correlation of the local level of synchronization. In this regard, when the slope is steeper, the information travels across shorter distances, while a flatter slope indicates that the information is transferred across longer distances. Thus, the negative slope stands for the spatial information transfer. Note that the parameter *A* depends on only *λ*. It depends neither on *t* as the correlation is over time, nor on the brain areas, as the pairwise correlations are organized as a function of the Euclidean distances.

#### Information cascade flow.

The information cascade flow characterizes the stream of information between a given scale (*λ*) and a subsequent lower scale (*λ* − Δ*λ*, where Δ*λ* is a scale step) in consecutive time steps (*t* and *t* + Δ*t*). In this way, the information cascade flow covers the information transfer across scales computed as the time correlation between the Kuramoto local order parameter in two consecutive scales and times:

$$𝓕\left(\lambda \right)={\langle {\text{corr}}_{t}\left(R_{\lambda }\left(\bar{x},t+\Delta t\right),R_{\lambda -\Delta \lambda }\left(\bar{x},t\right)\right)\rangle }_{\bar{x}},$$ (4)

where the brackets 〈
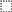
〉_$\bar{x}$_ denote averages across time and space, and *corr*_*t*_ refers to the pairwise time correlations.

#### Information cascade.

Finally, the information cascade is defined by averaging the information transfer across scales *λ*, capturing the entire information-processing behavior across scales.

#### Node variability of local synchronization.

The node variability of the local synchronization is defined as the standard deviation across time of the local Kuramoto order parameter, such as the following:

$$NLS\left(n,\lambda \right)={\langle R_{\lambda \left(n\right)}{\left(t\right)}^{2}\rangle }_{t}-{\langle R_{\lambda \left(n\right)}\left(t\right)\rangle }_{t}^{2},$$ (5)

where *n* is a node, and the brackets 〈
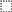
〉_*t*_ denote the average values across time points. Further, we used the discrete version of the node-level Kuramoto order parameter in Equation 2 (because we are computing a node-level metric over a given parcellation), with modulus *R*, indicating a spatial average of the complex phase factor of the local oscillators weighted by the coupling calculated through

$$R_{\lambda \left(n\right)}\left(t\right)=\left\|\sum_{p}\left[\frac{C_{\lambda \left(np\right)}}{\sum_{q}C_{\lambda \left(np\right)}}\right]e^{i\phi_{p}\left(t\right)}\right\|,$$ (6)

where ‖
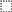
‖ is the modulus of the complex number, *ϕ*_*p*_(*t*) are the phases of the spatiotemporal data, *C*_*λ*(*np*)_ is the local weighting kernel between node *n* and *p*, and *λ* defines the spatial scaling:

$$C_{np}=e^{-\lambda \left(r\left(n,p\right)\right)},$$ (7)

where *r*(*n*, *p*) is the Euclidian distance between the brain areas *n* and *p* in MNI space.

### Model-Based Framework

#### Whole-brain computational model.

We built a whole-brain computational model where the local dynamics of each brain area (node) are described by the normal form of a supercritical Hopf bifurcation (also known as Stuart-Landau), which can describe the transition from noise-induced oscillations to fully sustained oscillations (Deco, Cabral, et al., 2017). This model is characterized by two parameters that rule the global dynamical behavior: the multiplicative factor, *G*, denoting the global conductivity of the fibers, scaling the structural connectivity between brain areas, which is assumed to be equal throughout the brain (Deco, Cabral, et al., 2017; Deco, Tagliazucchi, Laufs, Sanjuán, & Kringelbach, 2017); and the local bifurcation parameter (*a*_*j*_), which rules the dynamical behavior of each area between noise-induced, leading the system to a stable fixed point (*a* < 0), a stable limit cycle producing self-sustained oscillations (*a* > 0), or a critical behavior between both (*a* ∼ 0). We optimized the model parameters to better fit the empirical functional connectivity as a function of distance, *r*, within the inertial subrange. The model considered 1,000 cortical brain areas from the Schaefer atlas mentioned above. The underlying anatomical matrix *C*_*np*_ was added to link the brain structure and functional dynamics and was obtained by measuring the exponential distance rule as defined in Equation 2. The spontaneous local dynamics of each brain area were described by the normal form of a supercritical Hopf bifurcation, which simulates the dynamics for each brain area from noisy to oscillatory dynamics as follows:

$$\frac{dx_{n}}{dt}=a_{n}x_{n}-\left[x_{n}^{2}+y_{n}^{2}\right]x_{n}-\omega_{n}y_{n}+\nu \eta_{n}\left(t\right),$$ (8)

$$\frac{dy_{n}}{dt}=a_{n}y_{n}-\left[x_{n}^{2}+y_{n}^{2}\right]y_{n}+\omega_{n}x_{n}+\nu \eta_{n}\left(t\right),$$ (9)

where *η*_*n*_(*t*) is additive Gaussian noise with standard deviation *ν* = 0.01. The frequency *ω*_*n*_ of each brain area was determined from the empirical fMRI data as the peak of the power spectrum. This normal form has a supercritical bifurcation at *a*_*n*_ = 0, such that for *a*_*n*_ > 0 the system is in a stable limit cycle oscillation with frequency *f*_*n*_ = *ω*_*n*_/2*π*, whereas for *a*_*n*_ < 0 the local dynamics are in a stable point (i.e., noisy state). Lastly, the whole-brain dynamics were described by the following set of coupled equations:

$$\frac{dx_{n}}{dt}=a_{n}x_{n}-\left[x_{n}^{2}+y_{n}^{2}\right]x_{n}-\omega_{n}y_{n}+G\sum_{p=1}^{N}C_{np}\left(x_{p}\left(t\right)-x_{n}\right)+\nu \eta_{n}\left(t\right),$$ (10)

$$\frac{dy_{n}}{dt}=a_{n}y_{n}-\left[x_{n}^{2}+y_{n}^{2}\right]y_{n}+\omega_{n}x_{n}+G\sum_{p=1}^{N}C_{np}\left(y_{p}\left(t\right)-y_{p}\right)+\nu \eta_{n}\left(t\right),$$ (11)

where the global coupling factor *G*, scaled equally for each brain area, denotes the input received in a region *n* from every other region *p*.

#### Functional connectivity fitting.

Kolmogorov’s structure-function (Frisch, 1995; Kolmogorov, 1941a, 1941b) of a variable *u* was applied to the BOLD signal of the data. This measure is based on the functional correlations between each pair of brain areas with equal Euclidean distance and was defined as the following:

$$S\left(r\right)={\langle {\left(u\left(\bar{x}+r\right)-u\left(\bar{x}\right)\right)}^{2}\rangle }_{\bar{x},t}=2\left[FC\left(0\right)-FC\left(r\right)\right],$$ (12)

where *FC*(*r*) is the spatial correlations of two points separated by a Euclidean distance *r*, which is given by

$$FC\left(r\right)={\langle u\left(\bar{x}+r\right)u\left(\bar{x}\right)\rangle }_{\bar{x},t},$$ (13)

where the symbol 〈
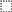
〉_$\bar{x}$,*t*_ refers to the average across the spatial location *x* of the brain areas and time, and assuming stationarity. Thus, the structure functions are characterizing the evolution of the functional connectivity as function of the Euclidean distance between equally distant nodes, which is different from the usual definition of FC that does not include distance. The simulated and the empirical *FC*(*r*) were compared using the Euclidian distance within the inertial range defined in Deco and Kringelbach (2020), allowing for adequate model fitting performance testing in describing the changes observed in the resting-state FC.

#### Susceptibility.

The susceptibility measure of the whole-brain model was defined as the brain’s sensitivity to react to external perturbations. The Hopf model was perturbed for each *G* by randomly changing the local bifurcation parameter, *a*_*n*_, in the range [−0.02:0]. The sensitivity of the perturbations on the spatiotemporal dynamics was calculated by measuring the modulus of the local Kuramoto order parameter as the following:

$$\chi ={\langle {\langle \left({\langle {\tilde{R}}_{\lambda_{s}}\left(\bar{x},t\right)\rangle }_{t}-{\langle R_{\lambda_{s}}\left(\bar{x},t\right)\rangle }_{t}\right)\rangle }_{\text{trials}}\rangle }_{\bar{x}},$$ (14)

where $\tilde{R}$_*λ*_*s*__($\bar{x}$, *t*) corresponds to the perturbed case, the *R*_*λ*_*s*__($\bar{x}$, *t*) to the unperturbed case, and 〈
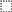
〉_*t*_, 〈
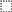
〉_*trials*_, and 〈
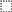
〉_$\bar{x}$_ to the average across time, trials, and space, respectively.

#### Information-encoding capability.

The information-encoding capability captures how the external perturbations are encoded in whole-brain dynamics. The information capability, *I*, was defined as the standard deviation across trials of the difference between the perturbed $\tilde{R}$_*λ*_*s*__($\bar{x}$, *t*) and unperturbed *R*_*λ*_*s*__($\bar{x}$, *t*) mean of the modulus of the local Kuramoto order parameter across time *t*, averaged across all brain areas *n* as the following:

$$I={\langle {\langle {\left({\langle {\tilde{R}}_{\lambda_{s}}\left(\bar{x},t\right)\rangle }_{t}-{\langle R_{\lambda_{s}}\left(\bar{x},t\right)\rangle }_{t}\right)}^{2}\rangle }_{\text{trials}}\rangle }_{\bar{x}}-{\langle {{\langle \left({\langle {\tilde{R}}_{\lambda_{s}}\left(\bar{x},t\right)\rangle }_{t}-{\langle R_{\lambda_{s}}\left(\bar{x},t\right)\rangle }_{t}\right)\rangle }^{2}}_{\text{trials}}\rangle }_{\bar{x}},$$ (15)

where the brackets 〈
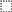
〉_*t*_, 〈
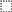
〉_*trials*_, and 〈
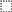
〉_$\bar{x}$_ denote the averages defined as above.

### Statistical Analyses

Differences between conditions in turbulence, information transfer, flow, and capability were statistically assessed using a permutation-based paired *t* test. This nonparametric test uses permutations of group labels to estimate the null distribution computed independently for each condition. We compared conditions employing a *t* test to each of the 1,000 permutations and used a significance threshold of *α* = 0.05. Additionally, we used the false discovery rate at the 0.05 level of significance to correct multiple comparisons (Hochberg & Benjamini, 1990). We applied the Wilcoxon rank sum method to compute the difference between conditions in susceptibility and information-encoding capacity measures. The similitude of the distributions of node-level turbulence per spatial scale, between each psychedelic drug and its counterpart placebo, was assessed using the Kolmogorov–Smirnov distance (KSD). The KSD quantifies the maximal difference between the cumulative distribution functions of the two samples.

## ACKNOWLEDGMENTS

The contents of this publication are solely the responsibility of the authors and do not represent the official views of the funding institutions.

## SUPPORTING INFORMATION

Supporting information for this article is available at https://doi.org/10.1162/netn_a_00250.

## AUTHOR CONTRIBUTIONS

Josephine Cruzat: Conceptualization; Formal analysis; Investigation; Methodology; Validation; Visualization; Writing – original draft; Writing – review & editing. Yonatan Sanz Perl: Conceptualization; Formal analysis; Methodology; Supervision; Validation; Visualization. Anira Escrichs: Software. Jakub Vohryzek: Validation. Christopher Timmermann: Validation. Leor Roseman: Data curation; Validation. Andrea I. Luppi: Data curation. Agustin Ibañez: Funding acquisition; Writing – review & editing. David Nutt: Data curation; Validation. Robin Carhart-Harris: Data curation; Funding acquisition; Supervision; Validation; Writing – review & editing. Enzo Tagliazucchi: Supervision; Validation; Writing – review & editing. Gustavo Deco: Conceptualization; Investigation; Methodology; Resources; Supervision; Validation; Writing – review & editing. Morten L. Kringelbach: Conceptualization; Investigation; Methodology; Resources; Supervision; Validation; Visualization; Writing – review & editing.

## FUNDING INFORMATION

Yonatan Sanz Perl, EU H2020 FET Flagship programme, Award ID: HBP SGA3 945539. Anira Escrichs, EU H2020 FET Flagship programme, Award ID: HBP SGA3 945539. Jakub Vohryzek, EU H2020 FET Proactive project Neurotwin, Award ID: 101017716. Andrea I. Luppi, Gates Cambridge scholarship. Agustin Ibañez, Takeda, CW2680521; CONICET; ANID/FONDECYT Regular (1210195 and 1210176); FONCYT-PICT 2017-1820; ANID/FONDAP/15150012; Sistema General de Regalías (BPIN2018000100059), Universidad del Valle (CI 5316); Programa Interdisciplinario de Investigación Experimental en Comunicación y Cognición (PIIECC), Facultad de Humanidades, USACH; Alzheimer’s Association GBHI ALZ UK-20-639295; and the Multi-Partner Consortium to Expand Dementia Research in Latin America [ReDLat, supported by National Institutes of Health, National Institutes of Aging (R01 AG057234), Alzheimer’s Association (SG-20-725707), Rainwater Charitable Foundation–Tau Consortium, and Global Brain Health Institute)]. Enzo Tagliazucchi, Mercator Fellowship granted by the German Research Foundation, ANPCyT, Award ID: PICT-2019-02294 and Award ID: PICT-2018-03103. Gustavo Deco, Spanish Ministry of Science, Innovation and Universities (MCIU), Award ID: PID2019-105772GB-I00 MCIU AEI), State Research Agency (AEI), EU H2020 FET Flagship programme, Award ID: HBP SGA3 945539, SGR Research Support Group support, Award ID: 2017 SGR 1545, funded by the Catalan Agency for Management of University and Research Grants (AGAUR), EU H2020 FET Proactive programme, Award ID: 101017716), EU H2020 MSCA-ITN Innovative Training Networks: euSNN European School of Network Neuroscience, Award ID: 860563, CECH The Emerging Human Brain Cluster, Award ID: 001-P-001682, within the framework of the European Research Development Fund Operational Program of Catalonia 2014–2020, Fundacio La Marato TV3, Brain-Connects: Brain Connectivity during Stroke Recovery and Rehabilitation, Award ID: 201725.33, FLAG–ERA JTC 2017, Spanish Ministry of Science, Innovation and Universities (MCIU), State Research Agency (AEI), Award ID: PCI2018-092891. Morten L. Kringelbach, Danish National Research Foundation, Award ID: DNRF117, Pettit and Carlsberg Foundations.

## Supplementary Material



## TECHNICAL TERMS

Turbulence: In coupled oscillators, turbulence is characterized by the high variability across space and time of the local Kuramoto parameter capturing the local synchronization, so in this sense it is a spatiotemporal extension of the level of metastability.
Functional hierarchy: General and intrinsic organizing principle of brain function that arises because of the progressive integration of information.
Brain state: Continuously evolving dynamics of self-organized, condition-dependent, system-wide patterns of synchronous neural activity.
Information transfer: A measure that characterizes how the information propagates across space at a particular scale.
Information cascade flow: A measure that estimates the stream of information between a given scale and a subsequent lower scale in consecutive time steps.
Information cascade: A measure that captures the entire efficient information-processing behavior across scales.
Metastability: Fundamental concept describing complex systems' behavior in terms of the variability in the global state of synchronization as a function of time.
