# Supplementary material for: Effects of classic psychedelic drugs on turbulent signatures in brain dynamics
Source: Netw Neurosci. 2022 Oct 1;6(4):1104–24. doi: 10.1162/netn_a_00250 (PMC11117113; doi:10.1162/netn_a_00250)
Supplement: Supplementary file 1 [file netn-6-4-1104-s001.pdf]

Cruzat, J., Perl, Y. S., Escrichs, A., Vohryzek, J., Timmermann, C., Roseman, L., Luppi, A. I., Ibañez, A., Nutt, D., Carhart-Harris, R., Tagliazucchi, E., Deco, G., & Kringelbach, M. L. (2022). Supporting information for "Effects of classic psychedelic drugs on turbulent signatures in brain dynamics." *Network Neuroscience*. Advance publication. http://doi.org/10.1162/netn_a_00250

SUPPLEMENTAL INFORMATION

Effects of classic psychedelic drugs on turbulent signatures in brain dynamics

Josephine Cruzat, Yonatan Sanz Perl, Anira Escrichs, Jakub Vohryzek, Christopher Timmermann, Leor Roseman, Andrea I. Luppi, Agustin Ibañez, David Nutt, Robin Carhart-Harris, Enzo Tagliazucchi, Gustavo Deco & Morten L. Kringelbach

SUPPLEMENTAL METHODS

Subjective ratings

For both LSD and Psilocybin experiments, in scanner, VAS ratings were obtained after each scan. The scales included items for intensity, simple imagery, complex imagery, positive mood and ego dissolution and emotional arousal. Specifically, they were phrased as follows: 1) "Please rate the intensity of the drug effects during the last scan", with a bottom anchor of "no effects", a mid-point anchor of "moderately intense effects" and a top anchor of "extremely intense effects"; 2) "With eyes closed, I saw patterns and colours", with a bottom anchor of "no more than usual" and a top anchor of "much more than usual"; 3) "With eyes closed, I saw complex visual imagery", with the same anchors as item 2; 4) "How positive was your mood for the last scan?", with the same anchors as item 2, plus a mid-point anchor of "somewhat more than usual"; 5) "I experienced a dissolving of my self or ego", with the same anchors as item 2; and 6) "Please rate your general level of emotional arousal for the last scan", with a bottom anchor of "not at all emotionally aroused", a mid-point anchor of "moderately emotionally aroused" and a top anchor of "extremely emotionally aroused".

SUPPLEMENTAL FIGURES AND LEGENDS

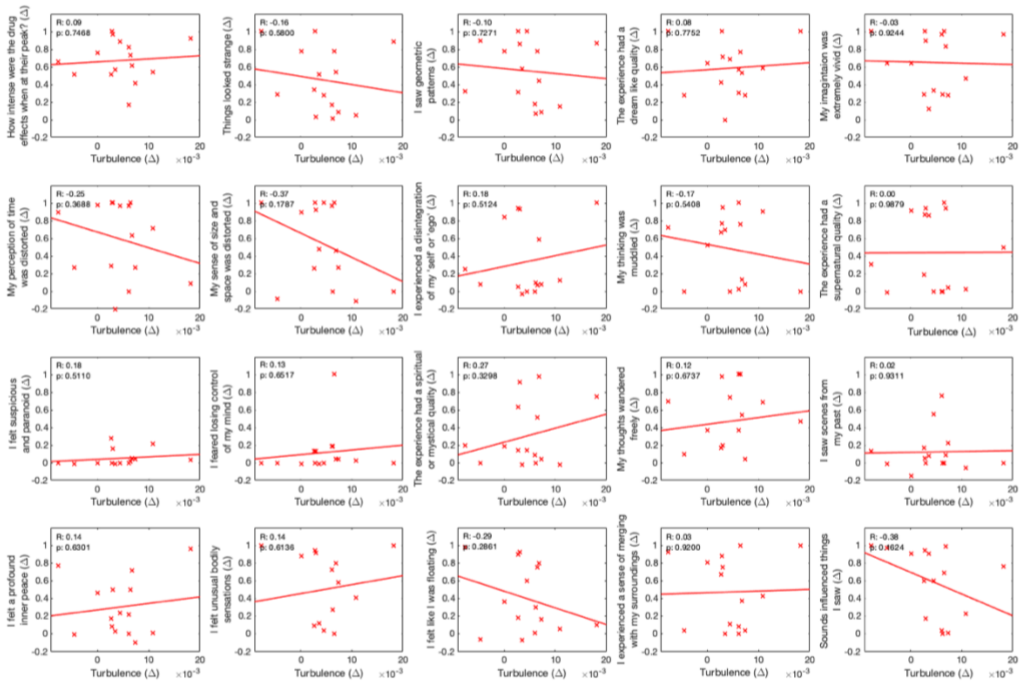

Figure S1.a. Correlation between turbulence under LSD minus placebo and different VAS scale items. In the inset, Pearson's R and the associated (uncorrected) p-values are provided. The increases in turbulence did not correlate with the subjective experience.

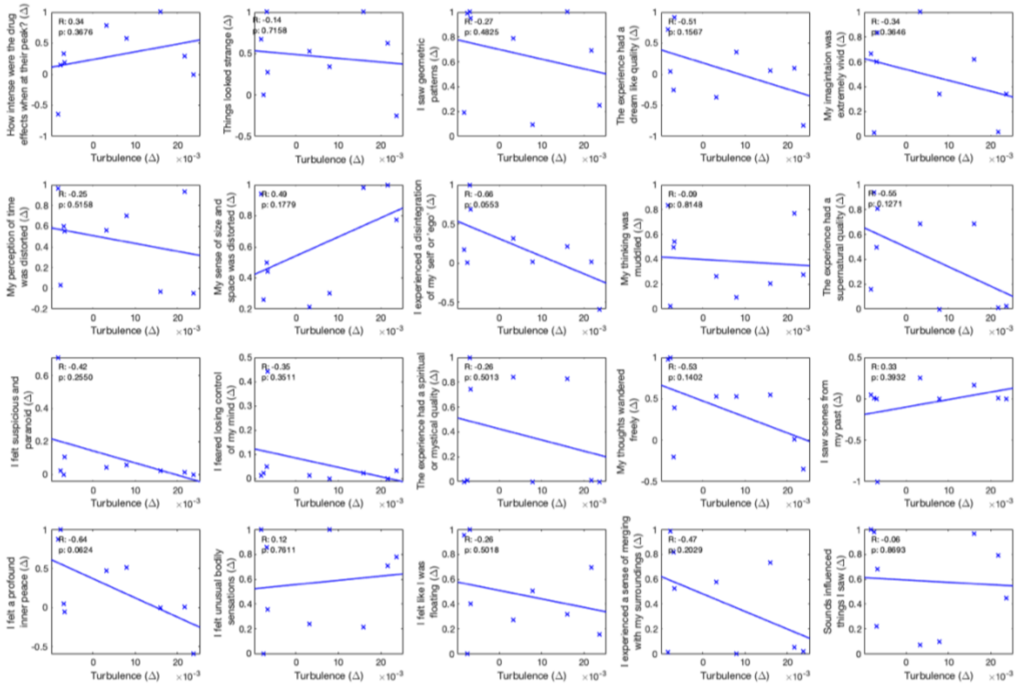

Figure S1.b Correlation between turbulence under psilocybin minus placebo and different VAS scale items. In the inset, Pearson's R and the associated (uncorrected) p-values are provided. The increases in turbulence did not correlate with the subjective experience under psilocybin.

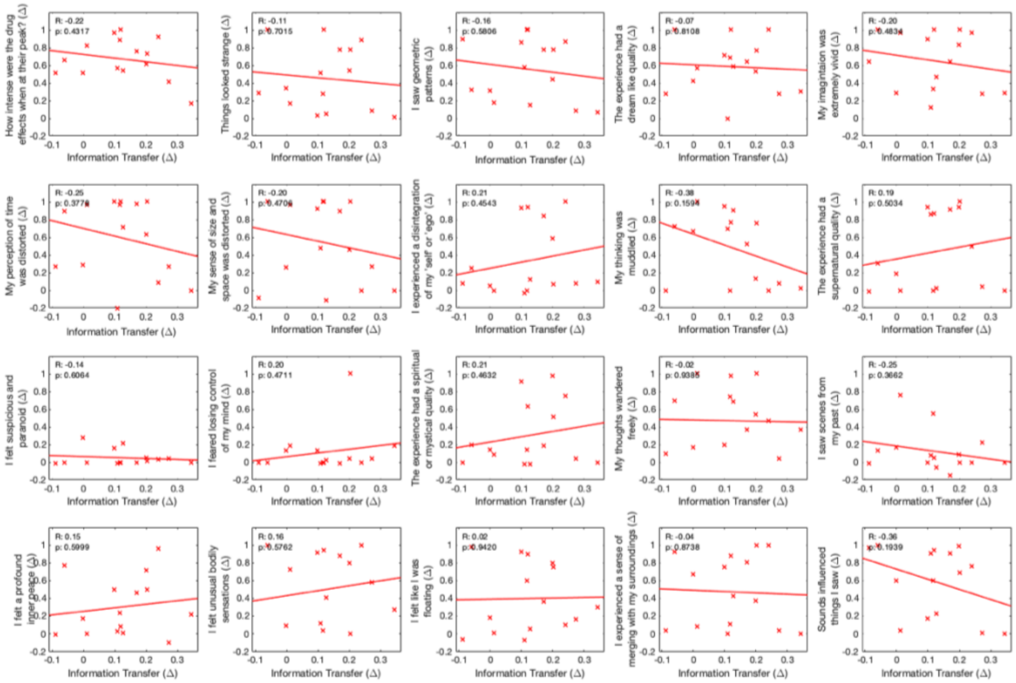

Figure S2.a Correlation between information transfer under LSD minus placebo and different VAS scale items. In the inset, Pearson's R and the associated (uncorrected) p-values are provided. The increases in information transfer did not correlate with the subjective experience under LSD.

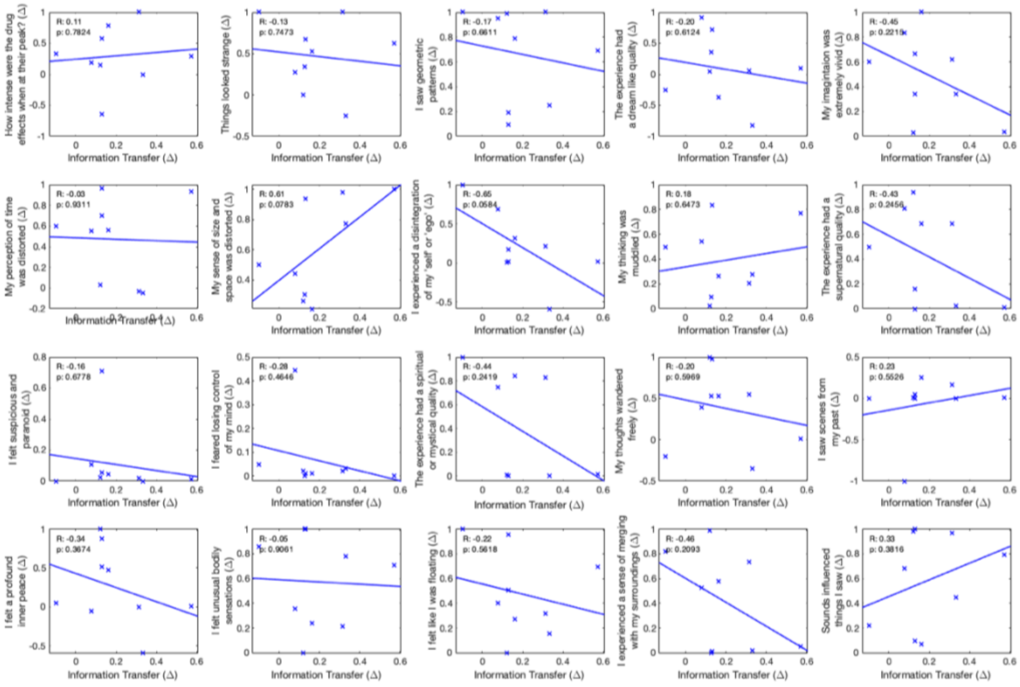

Figure S2.b Correlation between information transfer under psilocybin minus placebo and different VAS scale items. In the inset, Pearson's R and the associated (uncorrected) p-values are provided. The increases in information transfer did not correlate with the subjective experience under psilocybin.

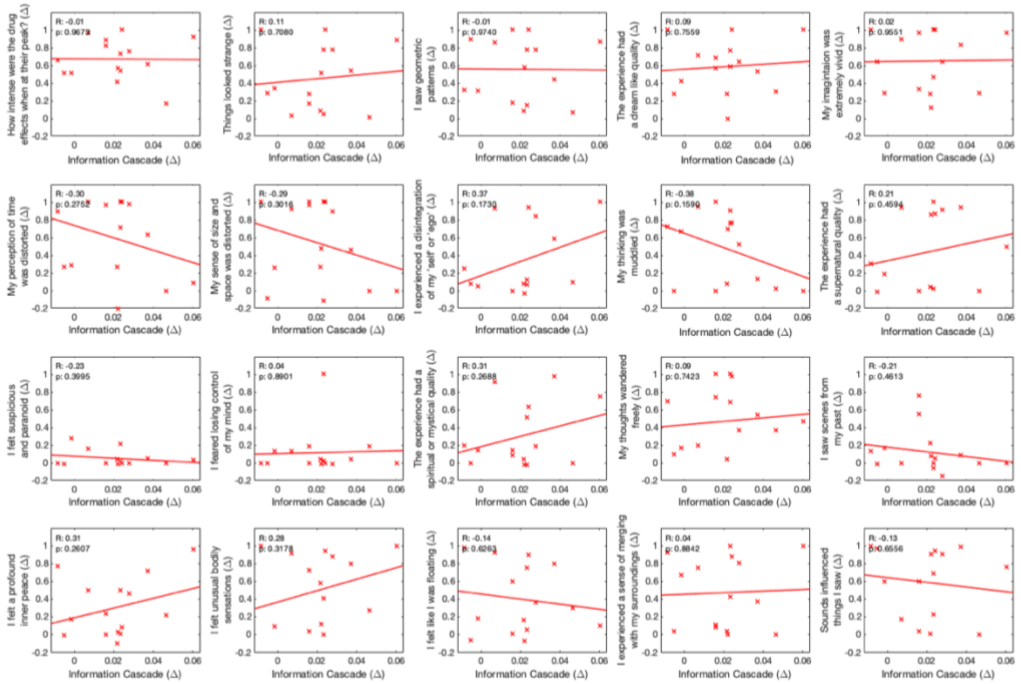

Figure S3.a Correlation between information cascade under LSD minus placebo and different VAS scale items. In the inset, Pearson's R and the associated (uncorrected) p-values are provided. The increases in information cascade did not correlate with the subjective experience under LSD.

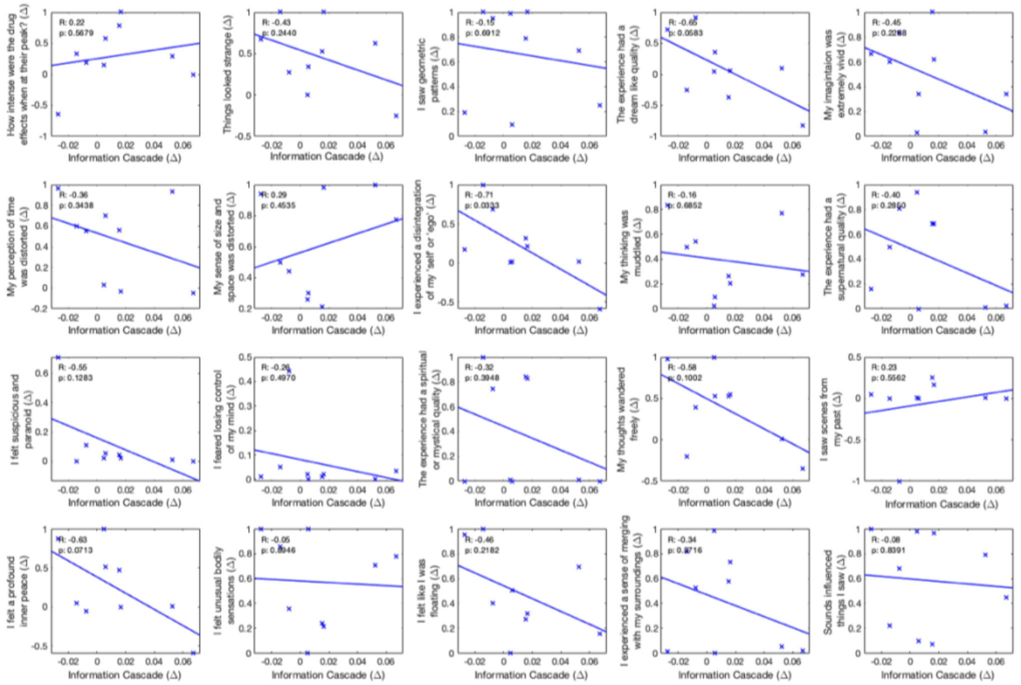

Figure S3.b Correlation between information cascade under psilocybin minus placebo and different VAS scale items. In the inset, Pearson's R and the associated (uncorrected) p-values are provided. The increases in information cascade correlate with the subjective experience of ego-dissolution under psilocybin.
